# Supplementary material for: A Likelihood Approach for Real-Time Calibration of Stochastic Compartmental Epidemic Models
Source: PLoS Comput Biol. 2017 Jan 17;13(1):e1005257. doi: 10.1371/journal.pcbi.1005257 (PMC5240920; doi:10.1371/journal.pcbi.1005257)
Supplement: S1 File — (TAR.GZ) [file pcbi.1005257.s014.tar.gz › HSPH_Online-SI-Revision/Additions/IPoi10/n100-extreme-SEITR/n100-extreme-SEITR_BENCH_table4.pdf]

1	R0	{1.38654, 9.85565}	{2.97157, 19.3547}	{{2.97157, 19.3547}, {2.97157, 19.3547}}	{114.315, 96.3818}	inf	602.	12 093.8	{5977., 23 554.}	154.58	0.355329	newly	2747.	3910.99	{1906., 7784.}	59.5339	0.186391	Reff	0.128602	0.319177	{0.227563, 0.494083}	148.19	ar	92 379.	93 833.9	{93 698., 93 960.}	1.57497
2	R0	{2.36629, 12.2029}	{2.9884, 12.8326}	{{2.9884, 12.8326}, {2.9884, 12.8326}}	{26.2905, 5.16025}	inf	104.	12 444.	{7040., 19 779.}	111.503	0.295881	newly	2664.	5665.64	{3171., 9061.}	112.978	0.295735	Reff	0.0301229	0.307336	{0.236263, 0.409112}	920.276	ar	99 022.	93 950.3	{93 806., 94 101.}	5.12181
3	R0	{2.32253, 12.836}	{2.9194, 10.1535}	{{2.9194, 10.1535}, {2.9194, 10.1535}}	{25.6991, 20.8983}	inf	185.	5185.41	{2490., 8240.}	43.9806	0.266676	newly	3413.	2782.06	{1321., 4511.}	41.0539	0.216778	Reff	0.035767	0.233904	{0.20973, 0.267213}	553.967	ar	98 997.	93 450.9	{93 299., 93 651.}	5.60226
4	R0	{1.79075, 11.2058}	{2.9884, 12.8326}	{{2.9884, 12.8326}, {2.9884, 12.8326}}	{66.8798, 14.5175}	inf	424.	8470.29	{4501., 13 755.}	43.5875	0.149578	newly	3304.	3818.04	{1962., 6232.}	39.2256	0.144579	Reff	0.0739401	0.256204	{0.212475, 0.315545}	246.502	ar	96 920.	93 979.9	{93 831., 94 135.}	3.03352
5	R0	{2.26952, 9.62371}	{2.98388, 9.60029}	{{2.98388, 9.60029}, {2.98388, 9.60029}}	{31.4763, 0.243357}	inf	39.	4978.6	{2534., 8859.}	102.788	0.256844	newly	1318.	2768.47	{1413., 5005.}	111.855	0.2731	Reff	0.0289591	0.219957	{0.196041, 0.252914}	659.543	ar	98 881.	93 934.1	{93 779., 94 075.}	5.00291
6	R0	{1.57191, 6.89004}	{2.91001, 10.5991}	{{2.91001, 10.5991}, {2.91001, 10.5991}}	{85.1257, 53.8322}	inf	100.	7805.06	{3587., 15 614.}	437.909	0.661588	newly	1057.	4121.72	{1853., 8336.}	289.945	0.515835	Reff	0.0825567	0.267369	{0.218251, 0.351849}	223.862	ar	95 046.	93 419.9	{93 254., 93 602.}	1.71083
7	R0	{1.99226, 17.2339}	{2.97157, 19.3547}	{{2.97157, 19.3547}, {2.97157, 19.3547}}	{49.1557, 12.306}	inf	602.	11 342.	{6507., 17 792.}	30.2165	0.133005	newly	3977.	3630.67	{2076., 5699.}	31.4413	0.148869	Reff	0.0686533	0.293951	{0.235259, 0.367851}	328.167	ar	97 992.	93 844.3	{93 685., 94 013.}	4.2327
8	R0	{2.63369, 15.1879}	{2.9884, 12.8326}	{{2.9884, 12.8326}, {2.9884, 12.8326}}	{13.4682, 15.5077}	inf	100.	11 945.1	{7523., 18 598.}	50.6881	0.158903	newly	3068.	5462.42	{3365., 8444.}	79.4536	0.226159	Reff	0.022123	0.331512	{0.23964, 0.398234}	1398.5	ar	99 451.	93 953.1	{93 809., 94 120.}	5.52826
9	R0	{2.53271, 9.90552}	{2.98388, 9.60029}	{{2.98388, 9.60029}, {2.98388, 9.60029}}	{17.8137, 3.08141}	inf	27.	5119.1	{2439., 9661.}	101.345	0.249154	newly	1330.	2861.93	{1372., 5448.}	116.71	0.275374	Reff	0.0191979	0.221518	{0.196459, 0.263089}	1053.87	ar	99 355.	93 939.7	{93 789., 94 099.}	5.45045
10	R0	{2.48791, 18.9003}	{2.7943, 13.5962}	{{2.7943, 13.5962}, {2.7943, 13.5962}}	{12.3152, 28.0636}	inf	387.	5678.37	{3023., 9588.}	62.1489	0.464738	newly	4707.	2442.95	{1299., 4169.}	50.9715	0.340027	Reff	0.0381148	0.262051	{0.235615, 0.300303}	587.532	ar	99 291.	92 444.9	{92 271., 92 617.}	6.89503
11	R0	{2.84414, 18.4765}	{2.86118, 15.5722}	{{2.86118, 15.5722}, {2.86118, 15.5722}}	{0.599127, 15.7189}	inf	161.	12 436.7	{7109., 21 337.}	33.5516	0.158363	newly	4255.	4858.79	{2699., 8618.}	37.8662	0.145225	Reff	0.0214164	0.339988	{0.255532, 0.488089}	1487.51	ar	99 654.	93 001.	{92 851., 93 158.}	6.6761
12	R0	{2.29369, 14.622}	{2.7943, 13.5962}	{{2.7943, 13.5962}, {2.7943, 13.5962}}	{21.8255, 7.01546}	inf	189.	12 107.9	{6982., 19 027.}	59.2025	0.180495	newly	3139.	5344.82	{3007., 8532.}	74.15	0.211236	Reff	0.0347723	0.358278	{0.271494, 0.468492}	930.354	ar	98 982.	92 454.6	{92 316., 92 606.}	6.59458
13	R0	{2.73413, 16.7529}	{2.99939, 13.7076}	{{2.99939, 13.7076}, {2.99939, 13.7076}}	{9.70181, 18.1777}	inf	128.	8217.07	{4467., 13 570.}	37.3195	0.197778	newly	3594.	3495.59	{1869., 5762.}	37.4008	0.166767	Reff	0.0212989	0.247842	{0.209147, 0.298049}	1063.64	ar	99 543.	94 055.	{93 911., 94 198.}	5.51322
14	R0	{1.79554, 16.5943}	{2.76795, 19.6041}	{{2.76795, 19.6041}, {2.76795, 19.6041}}	{54.157, 18.1376}	inf	1182.	11 031.9	{5968., 19 374.}	35.6658	0.183427	newly	4850.	3521.75	{1888., 6292.}	39.7852	0.221179	Reff	0.106063	0.343384	{0.272339, 0.484031}	223.756	ar	96 821.	92 233.	{92 088., 92 399.}	4.73861
15	R0	{1.91429, 13.7771}	{2.93978, 19.9817}	{{2.93978, 19.9817}, {2.93978, 19.9817}}	{53.5703, 45.0356}	inf	383.	13 444.5	{8082., 21 216.}	68.3953	0.201616	newly	3380.	4236.27	{2457., 6708.}	40.3272	0.141111	Reff	0.0667896	0.362754	{0.248353, 0.467454}	443.13	ar	97 541.	93 628.8	{93 471., 93 795.}	4.01079
16	R0	{1.42094, 10.5871}	{2.8939, 16.8013}	{{2.8939, 16.8013}, {2.8939, 16.8013}}	{103.661, 58.696}	inf	1054.	11 086.8	{6056., 21 062.}	60.0357	0.171695	newly	4015.	4068.36	{2171., 7734.}	46.0565	0.193378	Reff	0.134932	0.326508	{0.237647, 0.46641}	141.979	ar	93 044.	93 287.6	{93 133., 93 435.}	0.262361
17	R0	{1.45515, 6.16466}	{2.9194, 10.1535}	{{2.9194, 10.1535}, {2.9194, 10.1535}}	{100.625, 64.7049}	inf	170.	8463.03	{4508., 14 870.}	387.783	0.636305	newly	1376.	4655.82	{2396., 8152.}	238.359	0.470305	Reff	0.105091	0.279196	{0.223889, 0.340869}	165.671	ar	93 254.	93 469.1	{93 327., 93 615.}	0.233577
18	R0	{2.85669, 13.2416}	{2.83858, 10.6973}	{{2.83858, 10.6973}, {2.83858, 10.6973}}	{0.63395, 19.2144}	inf	31.	9017.52	{4865., 15 329.}	57.9438	0.172588	newly	2583.	4884.48	{2445., 8115.}	91.5989	0.228231	Reff	0.014512	0.366034	{0.244856, 0.475888}	2422.29	ar	99 644.	92 812.5	{92 663., 92 940.}	6.85588
19	R0	{2.56417, 17.9558}	{2.8939, 16.8013}	{{2.8939, 16.8013}, {2.8939, 16.8013}}	{12.8591, 6.42968}	inf	266.	16 618.2	{9843., 22 860.}	33.6554	0.126435	newly	4524.	6214.93	{3600., 9063.}	46.1441	0.154762	Reff	0.0335137	0.439399	{0.283834, 0.590819}	1211.1	ar	99 389.	93 282.	{93 172., 93 391.}	6.14453
20	R0	{2.27517, 11.1864}	{2.92375, 10.9639}	{{2.92375, 10.9639}, {2.92375, 10.9639}}	{28.5069, 1.98902}	inf	92.	8068.7	{4671., 13 859.}	87.8029	0.238883	newly	2013.	4114.24	{2382., 7179.}	104.98	0.272978	Reff	0.0313746	0.262449	{0.225509, 0.332518}	736.502	ar	98 894.	93 493.9	{93 362., 93 657.}	5.46049
21	R0	{2.35735, 10.4735}	{2.9194, 10.1535}	{{2.9194, 10.1535}, {2.9194, 10.1535}}	{23.8425, 3.05533}	inf	65.	8358.86	{4726., 14 655.}	124.871	0.309103	newly	1848.	4537.66	{2501., 8000.}	145.544	0.343214	Reff	0.0275103	0.268261	{0.22856, 0.341278}	875.131	ar	99 036.	93 474.8	{93 322., 93 640.}	5.6153
22	R0	{1.79551, 11.9179}	{2.7943, 13.5962}	{{2.7943, 13.5962}, {2.7943, 13.5962}}	{55.6271, 14.0822}	inf	355.	6029.08	{3041., 10 411.}	42.516	0.183493	newly	2882.	2604.92	{1319., 4561.}	43.8979	0.202214	Reff	0.0710663	0.27005	{0.233631, 0.310978}	279.997	ar	97 033.	92 426.9	{92 269., 92 561.}	4.74693
23	R0	{1.23545, 6.31687}	{2.9884, 12.8326}	{{2.9884, 12.8326}, {2.9884, 12.8326}}	{141.888, 103.148}	inf	264.	8615.74	{4683., 13 435.}	419.333	0.672815	newly	1370.	3876.48	{2047., 6065.}	182.954	0.406813	Reff	0.148353	0.257528	{0.215583, 0.30404}	73.5913	ar	88 739.	93 936.	{93 810., 94 049.}	5.85654
24	R0	{2.24089, 10.1789}	{2.83858, 10.6973}	{{2.83858, 10.6973}, {2.83858, 10.6973}}	{26.672, 5.09289}	inf	88.	9314.11	{4523., 15 810.}	123.846	0.298419	newly	2182.	4946.94	{2307., 8429.}	128.643	0.301738	Reff	0.0325825	0.30513	{0.243919, 0.380455}	836.482	ar	98 868.	92 823.4	{92 660., 92 988.}	6.1138
25	R0	{1.57298, 10.2607}	{2.86118, 15.5722}	{{2.86118, 15.5722}, {2.86118, 15.5722}}	{81.8955, 51.7655}	inf	633.	15 563.1	{10 041., 20 405.}	137.279	0.353394	newly	3489.	6155.59	{3810., 8049.}	78.8505	0.231774	Reff	0.10572	0.407767	{0.287949, 0.473354}	285.705	ar	94 957.	93 012.4	{92 823., 93 214.}	2.04783
26	R0	{1.2154, 8.2576}	{2.8939, 16.8013}	{{2.8939, 16.8013}, {2.8939, 16.8013}}	{138.103, 103.465}	inf	684.	13 896.1	{8126., 20 860.}	258.61	0.522644	newly	2648.	5130.53	{2950., 7737.}	94.0874	0.251669	Reff	0.165416	0.370426	{0.262506, 0.482876}	123.936	ar	88 421.	93 274.4	{93 083., 93 472.}	5.48893
27	R0	{2.22841, 18.7983}	{2.8939, 16.8013}	{{2.8939, 16.8013}, {2.8939, 16.8013}}	{29.8639, 10.6233}	inf	454.	7178.21	{3652., 11 305.}	51.5433	0.329516	newly	4125.	2613.38	{1329., 4040.}	48.4441	0.289144	Reff	0.0518997	0.274236	{0.223409, 0.298506}	428.396	ar	98 756.	93 268.2	{93 094., 93 424.}	5.55693
28	R0	{1.27778, 5.36767}	{2.9194, 10.1535}	{{2.9194, 10.1535}, {2.9194, 10.1535}}	{128.474, 89.1603}	inf	160.	5965.06	{3042., 9023.}	435.945	0.6703	newly	965.	3232.11	{1640., 4919.}	235.07	0.463381	Reff	0.136275	0.243508	{0.212211, 0.271446}	78.6884	ar	89 843.	93 463.3	{93 309., 93 612.}	4.02956
29	R0	{2.38042, 15.8901}	{2.99939, 13.7076}	{{2.99939, 13.7076}, {2.99939, 13.7076}}	{26.0026, 13.735}	inf	266.	5228.69	{2964., 8483.}	55.9385	0.391545	newly	4278.	2206.9	{1203., 3549.}	50.0736	0.33296	Reff	0.0399673	0.218007	{0.19679, 0.24568}	445.465	ar	99 112.	94 052.7	{93 911., 94 204.}	5.10461
30	R0	{2.53188, 12.7607}	{2.9194, 10.1535}	{{2.9194, 10.1535}, {2.9194, 10.1535}}	{15.3056, 20.4315}	inf	140.	8357.07	{4419., 15 332.}	38.3065	0.15745	newly	3519.	4645.99	{2361., 8439.}	50.2926	0.158338	Reff	0.0262556	0.295915	{0.22386, 0.347087}	1027.06	ar	99 317.	93 469.3	{93 307., 93 623.}	5.88791
31	R0	{1.24482, 10.5471}	{2.86095, 19.9929}	{{2.86095, 19.9929}, {2.86095, 19.9929}}	{129.828, 89.5583}	inf	1178.	13 483.3	{7303., 23 101.}	124.871	0.317921	newly	3288.	4254.08	{2264., 7390.}	44.9288	0.154238	Reff	0.174511	0.361354	{0.258573, 0.536171}	107.066	ar	89 097.	93 017.1	{92 854., 93 178.}	4.39984
32	R0	{2.30446, 17.9607}	{2.7943, 13.5962}	{{2.7943, 13.5962}, {2.7943, 13.5962}}	{21.2562, 24.3003}	inf	386.	10 487.2	{5355., 21 694.}	45.387	0.239043	newly	4308.	4684.68	{2286., 9833.}	49.4329	0.191846	Reff	0.0453748	0.357076	{0.257802, 0.52762}	686.948	ar	99 028.	92 461.3	{92 288., 92 628.}	6.63114
33	R0	{2.31579, 11.8236}	{2.98388, 9.60029}	{{2.98388, 9.60029}, {2.98388, 9.60029}}	{28.8493, 18.804}	inf	79.	1415.7	{706., 2452.}	67.7444	0.546233	newly	2012.	778.556	{388., 1391.}	61.904	0.470144	Reff	0.0280905	0.190257	{0.182285, 0.198577}	577.299	ar	99 012.	93 949.6	{93 792., 94 116.}	5.11296
34	R0	{1.6678, 10.6744}	{2.96307, 14.3219}	{{2.96307, 14.3219}, {2.96307, 14.3219}}	{77.6634, 34.1705}	inf	325.	8726.15	{4795., 14 232.}	89.186	0.239991	newly	2446.	3641.86	{1877., 5829.}	60.2561	0.179953	Reff	0.0850078	0.279845	{0.216274, 0.323745}	229.199	ar	95 809.	93 778.4	{93 641., 93 912.}	2.11945
35	R0	{1.65476, 7.07559}	{2.9194, 10.1535}	{{2.9194, 10.1535}, {2.9194, 10.1535}}	{76.4244, 43.5004}	inf	106.	8076.61	{4587., 13 466.}	378.756	0.639723	newly	1151.	4370.	{2461., 7274.}	279.67	0.536398	Reff	0.0754074	0.263337	{0.225582, 0.318244}	249.218	ar	95 746.	93 456.	{93 315., 93 593.}	2.39174
36	R0	{2.71807, 18.5959}	{2.95376, 15.278}	{{2.95376, 15.278}, {2.95376, 15.278}}	{8.67123, 17.8421}	inf	279.	17 444.7	{10 053., 26 211.}	32.0842	0.12758	newly	5029.	7111.52	{4016., 10 811.}	50.048	0.162342	Reff	0.0291921	0.452695	{0.274109, 0.643683}	1450.75	ar	99 540.	93 748.5	{93 603., 93 894.}	5.81824
37	R0	{2.6202, 12.7552}	{2.99939, 13.7076}	{{2.99939, 13.7076}, {2.99939, 13.7076}}	{14.4718, 7.46676}	inf	74.	13 330.3	{7429., 22 579.}	101.313	0.267787	newly	2840.	5792.73	{3127., 10 030.}	106.183	0.27345	Reff	0.0220097	0.32732	{0.235722, 0.461096}	1387.16	ar	99 426.	94 042.2	{93 935., 94 180.}	5.41493
38	R0	{1.47672, 9.81651}	{2.86118, 15.5722}	{{2.86118, 15.5722}, {2.86118, 15.5722}}	{93.7524, 58.6328}	inf	503.	9498.65	{4763., 16 339.}	105.861	0.26636	newly	2643.	3702.44	{1815., 6404.}	56.2661	0.172096	Reff	0.109425	0.305829	{0.239738, 0.381338}	179.488	ar	93 815.	93 006.	{92 857., 93 121.}	0.862387
39	R0	{1.90004, 12.3313}	{2.7943, 13.5962}	{{2.7943, 13.5962}, {2.7943, 13.5962}}	{47.0653, 10.2576}	inf	369.	9607.49	{4940., 16 303.}	48.7342	0.163498	newly	3317.	4187.94	{2099., 7225.}	47.4573	0.164803	Reff	0.0681354	0.314289	{0.2528, 0.399557}	361.272	ar	97 413.	92 441.6	{92 268., 92 613.}	5.10342
40	R0	{2.64218, 18.5237}	{2.9884, 12.8326}	{{2.9884, 12.8326}, {2.9884, 12.8326}}	{13.1036, 30.7233}	inf	174.	3614.71	{1955., 5573.}	68.9401	0.553759	newly	3674.	1598.86	{847., 2483.}	59.0594	0.419126	Reff	0.0267917	0.206704	{0.191915, 0.224638}	671.521	ar	99 474.	93 976.6	{93 817., 94 181.}	5.5265
41	R0	{1.6945, 7.17996}	{2.9194, 10.1535}	{{2.9194, 10.1535}, {2.9194, 10.1535}}	{72.2868, 41.4144}	inf	141.	7024.38	{4374., 9517.}	182.103	0.420675	newly	1635.	3779.34	{2341., 5121.}	131.153	0.333135	Reff	0.0694067	0.24946	{0.220911, 0.271679}	259.418	ar	96 332.	93 485.3	{93 344., 93 653.}	2.95513
42	R0	{1.84559, 18.0074}	{2.97157, 19.3547}	{{2.97157, 19.3547}, {2.97157, 19.3547}}	{61.0092, 7.48192}	inf	1048.	12 123.5	{7294., 19 234.}	30.4955	0.146974	newly	4650.	3893.06	{2297., 6294.}	32.4405	0.163319	Reff	0.093313	0.304913	{0.242213, 0.411295}	226.764	ar	97 358.	93 844.6	{93 699., 93 991.}	3.60875
43	R0	{2.24965, 17.0361}	{2.7943, 13.5962}	{{2.7943, 13.5962}, {2.7943, 13.5962}}	{24.2104, 20.1918}	inf	520.	6957.29	{3206., 12 400.}	53.5976	0.36649	newly	4598.	3002.34	{1349., 5366.}	47.6073	0.291473	Reff	0.0537891	0.279214	{0.236649, 0.333276}	419.09	ar	98 770.	92 443.	{92 292., 92 575.}	6.40582
44	R0	{2.54324, 15.7967}	{2.7943, 13.5962}	{{2.7943, 13.5962}, {2.7943, 13.5962}}	{9.87166, 13.9301}	inf	127.	12 305.2	{6777., 21 111.}	49.7804	0.161331	newly	3246.	5467.29	{2912., 9568.}	73.5348	0.207488	Reff	0.0255087	0.374256	{0.271774, 0.525384}	1367.17	ar	99 354.	92 432.5	{92 223., 92 609.}	6.96646
45	R0	{2.54943, 14.8032}	{2.9884, 12.8326}	{{2.9884, 12.8326}, {2.9884, 12.8326}}	{17.2184, 13.312}	inf	120.	11 713.4	{6704., 22 070.}	51.5019	0.156123	newly	3187.	5368.88	{3012., 10 375.}	71.4856	0.195925	Reff	0.0250609	0.305702	{0.232318, 0.459108}	1119.84	ar	99 380.	93 959.1	{93 826., 94 102.}	5.45468
46	R0	{2.37755, 12.6461}	{2.9194, 10.1535}	{{2.9194, 10.1535}, {2.9194, 10.1535}}	{22.7903, 19.7104}	inf	129.	3078.23	{1470., 5442.}	62.3388	0.459495	newly	3124.	1644.83	{766., 2913.}	56.6606	0.381607	Reff	0.0309795	0.214189	{0.198402, 0.233318}	591.39	ar	99 096.	93 476.2	{93 347., 93 623.}	5.67108
47	R0	{1.69279, 11.6636}	{2.95376, 15.278}	{{2.95376, 15.278}, {2.95376, 15.278}}	{74.4906, 30.9887}	inf	535.	9689.78	{4931., 15 923.}	44.4276	0.148307	newly	3657.	3805.78	{1888., 6190.}	35.3033	0.144206	Reff	0.0899041	0.280114	{0.218785, 0.356785}	211.569	ar	96 214.	93 702.9	{93 542., 93 872.}	2.60993
48	R0	{1.82402, 10.0038}	{2.9884, 12.8326}	{{2.9884, 12.8326}, {2.9884, 12.8326}}	{63.8359, 28.2773}	inf	312.	9109.52	{4505., 17 413.}	79.9233	0.208459	newly	2763.	4178.66	{2019., 8001.}	65.4313	0.183272	Reff	0.0676164	0.279938	{0.215523, 0.366079}	314.008	ar	97 076.	93 937.4	{93 788., 94 069.}	3.23315
49	R0	{1.6424, 11.1667}	{2.99939, 13.7076}	{{2.99939, 13.7076}, {2.99939, 13.7076}}	{82.6224, 22.7543}	inf	368.	4375.17	{1939., 8131.}	45.3012	0.216498	newly	2454.	1836.5	{818., 3453.}	47.0638	0.263616	Reff	0.0891166	0.212875	{0.192501, 0.24796}	138.873	ar	95 546.	94 023.4	{93 887., 94 157.}	1.59359
50	R0	{2.05638, 14.3048}	{2.99939, 13.7076}	{{2.99939, 13.7076}, {2.99939, 13.7076}}	{45.8578, 4.17482}	inf	362.	8574.6	{4878., 13 824.}	38.5747	0.178554	newly	3752.	3704.13	{2013., 5935.}	41.0329	0.176207	Reff	0.0564271	0.272967	{0.209267, 0.304618}	383.752	ar	98 194.	94 054.5	{93 915., 94 229.}	4.21561
